# Th2-High Severe Asthma with Hypereosinophilia in the Spectrum of Type 2 Inflammatory Diseases

**DOI:** 10.3390/ijms26115342

**Published:** 2025-06-02

**Authors:** Elizabeth Malaya, Kamil Marszałek, Piotr Kuna, Maciej Kupczyk, Michał Panek

**Affiliations:** Clinic of Internal Medicine, Asthma and Allergy, Medical University of Lodz, Kopcińskiego 22, 90-153 Lodz, Poland; elizabeth.malaya@stud.umed.lodz.pl (E.M.); kamil.marszalek@stud.umed.lodz.pl (K.M.); piotr.kuna@umed.lodz.pl (P.K.); maciej.kupczyk@umed.lodz.pl (M.K.)

**Keywords:** severe eosinophilic asthma, hypereosinophilia, eosinophilic disorders, type 2 inflammation, HES

## Abstract

Asthma is among the most common chronic respiratory diseases, affecting approximately 3340 individuals per 100,000 worldwide. It is a heterogeneous condition associated with airway hyperresponsiveness and chronic inflammation. Severe asthma (SA) affects 3–10% of patients, most of whom exhibit Type 2 (T2) inflammation with elevated eosinophil counts or increased fractional exhaled nitric oxide. Although the Global Initiative for Asthma provides detailed guidelines for SA, patients with marked hypereosinophilia (HE; >1500 cells/µL) who do not meet diagnostic criteria for hypereosinophilic syndrome (HES) or eosinophilic granulomatosis with polyangiitis (EGPA) remain insufficiently addressed. In such cases, oral corticosteroids, and T2-targeted monoclonal antibodies (MAbs) inhibiting interleukin-5 or its receptor are the main therapeutic options. For instance, mepolizumab is approved for EGPA, HES, and chronic rhinosinusitis with nasal polyps, but its use in hypereosinophilic SA is limited by eligibility, tolerance, or effectiveness. SA with HE not classified as HES or EGPA is exceptionally rare and may be diagnosed by the exclusion of other potential causes of HE. This review analyzes recent studies and case reports, aiming to expand the understanding of this underrecognized clinical entity, its relation to T2 inflammation and eosinophilic disorders, and to highlight the need for improved diagnostic and therapeutic strategies.

## 1. Introduction

Asthma is a heterogeneous condition typically associated with airway hyperresponsiveness and chronic inflammation [1]. It is one of the most common chronic respiratory diseases, affecting approximately 3340 individuals per 100,000 worldwide and responsible for over 450,000 deaths annually [1,2,3]. Approximately 3–10% of asthma patients have severe asthma (SA), which is diagnosed when symptoms persist despite optimal therapy and good adherence [1]. Type 2 (T2) inflammation characterizes approximately 60% of these cases, allowing differentiation between T2-high (allergic/atopic or non-allergic/eosinophilic) and T2-low asthma endotypes [1,4,5,6].

SA accompanied by hypereosinophilia (HE) represents a rare clinical entity, identified in approximately 0.3% of patients with asthma [7]. Eosinophilia is defined as a peripheral blood eosinophil (EOS) count exceeding 500 cells/µL, while HE refers to levels above 1500 cells/µL [8]. If this elevated EOS count is observed in samples taken more than one month apart, and other criteria, such as tissue infiltration and organ damage are met, the patient can be diagnosed as hypereosinophilic syndrome (HES) [9]. Another SA- and HE-associated condition relevant to treatment planning is eosinophilic granulomatosis with polyangiitis (EGPA) [1,10]. Only a limited number of cases of SA with HE have been reported in the literature. Therapeutic options remain unclear for patients who fail to meet the eligibility criteria for biologic treatment (with monoclonal antibodies [MAbs]), cannot tolerate it well, decline such therapy, or do not fulfill all diagnostic criteria for either HES or EGPA at the time of evaluation. Such cases remain underrepresented and insufficiently described in the literature to date.

The aim of this review is to analyze case reports and studies presenting SA associated with HE not classified as HES or EGPA—as these entities are already addressed in detail by condition-specific guidelines—and to compare them with recent findings on Type 2 inflammation and eosinophilic disorders reported within the last six years, potentially allowing for a more contextualized, up-to-date, and critically refined understanding of this clinical subset. Given the rarity of these presentations and their limited inclusion in clinical trials, further investigation is needed to refine current knowledge and improve reporting standards.

## 2. T2-High Asthma

T2-high asthma is mediated either by the adaptive immune response to allergens—resulting in interleukin (IL)-4, IL-5, and IL-13 production—or by innate immune activation through stimuli such as viruses, bacteria, or irritants, which trigger the release of IL-33, IL-25, and thymic stromal lymphopoietin (TSLP) from airway epithelial cells [1]. This immunologic cascade promotes the accumulation of eosinophils, mast cells, basophils, T helper (Th)2 cells (predominant in atopic asthma), and type 2 innate lymphoid cells (ILC2s). ILC2s are more prevalent in non-atopic asthma [11,12,13].

The allergic endotype typically manifests during childhood or adolescence (early-onset) and is associated with atopy, excessive immunoglobulin (Ig)E production in response to allergens, and activation of Th2 cells. IgE mediates both early- and late-phase allergic responses, contributing to bronchoconstriction and mucus hypersecretion, with IL-4 and IL-13 serving as key links to T2 inflammation [5]. Additionally, p38 mitogen-activated protein kinase (MAPK) induces differentiation and activation of Th2 cells, promoting the release of IL-4, -5, -13, thereby contributing to the inflammatory response [14,15]. Atopic asthma is frequently associated with a personal or family history of allergic diseases, becoming part of the “atopic march”, a progression of allergic conditions beginning in infancy. This sequence usually begins with atopic dermatitis (AD), followed by IgE-mediated food allergy (FA), asthma, and allergic rhinitis (AR). According to the newest research, eosinophilic esophagitis (EoE) is also now recognized as part of this sequence [1,16]. Induced sputum analysis in patients with allergic asthma often reveals eosinophilic airway inflammation, and patients with this phenotype generally respond well to inhaled corticosteroids (ICSs) [1].

In contrast to the allergic endotype, adult-onset eosinophilic asthma is marked by persistent eosinophilic airway inflammation that remains ICS-resistant from the outset, leading to frequent symptoms and severe exacerbations, often requiring maintenance oral corticosteroids (OCSs) [13]. This variant is often linked to chronic conditions such as chronic rhinosinusitis with nasal polyps (CRSwNP), with or without sensitivity to cyclooxygenase (COX-)1 inhibitors (non-steroidal anti-inflammatory drug [NSAID]-exacerbated airway disease [N-ERD]), or EGPA [6,13,17]. Late-onset eosinophilic asthma demonstrates a markedly higher level of eosinophil tissue infiltration than the allergic phenotype, with rates of 63% and 36%, respectively [13].

In differentiating T2-high from T2-low asthma, both parameters—fractional exhaled nitric oxide (FeNO) and blood EOS counts—play a crucial role. Other important markers include sputum EOS levels and clinically allergen-driven asthma [1,5]. FeNO is not a diagnostic tool for asthma in general, as it has low specificity and can be affected by other conditions such as smoking or bronchoconstriction. FeNO correlates modestly with EOS levels but is unreliable in obese or Th2-low patients [1]. It remains the least invasive biomarker for classifying T2-high asthma, predicting responses to ICSs or biologics, and helping to identify poor adherence to therapy [1]. Elevated blood EOS count is linked to more frequent exacerbations and lung function decline, with the European Respiratory Society (ERS) and American Thoracic Society (ATS) recommending blood EOS counts of 150–300 cells/µL and sputum eosinophil thresholds of 2–3% as diagnostic markers of eosinophilic asthma [5]. While elevated serum IgE is common in asthma, significantly higher levels in T2-high asthma suggest its potential role as a biomarker for eosinophilic allergic asthma in adults [5]. Serum IL-37 may be elevated in allergic airway inflammation, where it downregulates the amplifying effects of IL-1β and IL-33, but like FeNO, it has low specificity and might be affected by other conditions such as psoriasis, arthritis, AR, and systemic lupus erythematosus [18,19].

Asthma has no cure; therapy aims to improve symptom control and reduce the risk of future exacerbations. The approach recommended by the Global Initiative for Asthma (GINA) is referred to as maintenance and reliever therapy (MART or SMART, for single inhaler use). This strategy relies heavily on the regular use of ICSs in combination with a long-acting β2-agonist (LABA), preferably formoterol [1]. Six monoclonal antibodies (MAbs) have been approved to treat T2-high SA, with proven safety and efficacy in clinical trials: dupilumab (anti-IL-4Rα), mepolizumab (anti-IL-5), omalizumab (anti-IgE), reslizumab, benralizumab (anti-IL-5Rα), and tezepelumab (anti-TSLP) [1]. Notably, mepolizumab may be indicated to treat EGPA, HES, and CRSwNP [1,20].

### 2.1. Hypereosinophilia and T2-High Asthma

Eosinophils are primarily tissue-resident cells with limited blood circulation under steady-state conditions, normally found in the thymus, adipose tissue, gastrointestinal tract (which contains the highest count, 20–30% of the total number of resident leukocytes), lungs, and female reproductive system [21]. Eosinophil production in the bone marrow is regulated by transcription factors and cytokines, primarily IL-5, IL-3, and granulocyte-macrophage colony-stimulating factor (GM-CSF), which are produced by activated T cells, stromal cells, and mast cells. Th2 cell-mediated immune responses and IL-5 production stimulate eosinophilopoiesis and activate eosinophils; when this activation is excessive or prolonged, eosinophils may migrate into non-native tissues such as the skin, heart, and lungs [22,23,24]. At these sites, activated eosinophils undergo degranulation, releasing cationic proteins and proteolytic enzymes, which contribute to eosinophilia and tissue damage, primarily through the induction of thrombosis and fibrosis [23].

When the EOS count exceeds 500 cells/µL in peripheral blood, eosinophilia or hypereosinophilia may be diagnosed. Eosinophilia is classified as mild (with an eosinophil count of <1500 cells/µL), moderate (1500–5000 cells/µL), or severe (>5000 cells/µL). The threshold for HE serves primarily as a risk marker rather than a definitive predictor of clinical severity. End organ damage may occur in the setting of only moderate eosinophilia, whereas, in rare instances, markedly severe HE may remain asymptomatic [25]. Hypereosinophilia is frequently associated with immune dysregulation and immunodeficiency syndromes, often presented in childhood with dermatological manifestations, atopy, recurrent infections, and an increased risk of malignancies like neoplastic disorders [26]. Individuals with an elevated EOS count may remain asymptomatic for years or, conversely, may present with early-onset clinical manifestations involving one or multiple organs simultaneously [27]. They may also present with constitutional symptoms such as low-grade fever, night sweats, fatigue, and weight loss. These symptoms are frequently observed in conditions like myeloproliferative (MPN) and lymphoid neoplasms, EGPA, and drug reaction with eosinophilia and systemic symptoms (DRESS) syndrome [23].

The respiratory system is the second most commonly affected after the skin. In patients referred for evaluation of unexplained hypereosinophilia, respiratory complaints are present in approximately 35% of cases; dermatologic involvement occurs in about 45% [27]. Diagnostic evaluation of eosinophilia with pulmonary involvement presents a major clinical challenge due to the broad differential and overlapping clinical, laboratory, and radiologic features, particularly when differentiating eosinophilic asthma from other Type 2 inflammation-driven conditions, primary disorders with asthma-like presentations, or coexisting primary or idiopathic hypereosinophilia [28]. Although isolated respiratory symptoms are infrequently observed with hematological disorders, asthma may constitute one of their clinical manifestations as well [7]. A case described by Johnstone et al. illustrates this diagnostic complexity: A patient with chronic eosinophilic leukemia (CEL) initially presented with progressive shortness of breath, cough, and paroxysmal wheezing. Although asthma was suspected and treated with short-acting β2-agonists (SABAs) and ICSs, the clinical response was only moderate. Further investigation was prompted by a markedly elevated blood EOS count, constituting 70% of total white blood cells (WBCs), leading to the diagnosis of the primary cause masked by asthma [29]. In such cases, as well as many others, a structured and methodical diagnostic approach is warranted to guide appropriate therapeutic decisions [28]. Identifying the potential cause, however, may not be sufficient to guide therapeutic decision-making; for instance, Tabeze et al. reported the presence of the Janus kinase (JAK)2 V617F mutation in 3 out of 34 patients with severe asthma and peripheral hypereosinophilia. All three presented with early-onset atopic asthma exacerbating in adulthood, but only one was diagnosed with a MPN based on bone marrow biopsy and received targeted treatment. The remaining two, with normal biopsy results, were managed with low-dose OCSs (e.g., prednisone) [7]. The JAK2 V617F point mutation, though rare, is implicated in the pathogenesis of hypereosinophilic states associated with MPNs such as polycythemia vera (PV) and essential thrombocythemia (ET), and was identified in 4% of cases in the German Registry study of 426 patients with hypereosinophilia of unknown significance, alongside 3% with a mutation in the KIT gene encoding the tyrosine kinase receptor (D816V) [7,24].

Considering all these non-asthma-related causes of eosinophilia before initiating treatment is essential for appropriate asthma management, as overlooking them may reduce therapeutic effectiveness; for example, in cases of undiagnosed parasitic infections, the use of oral corticosteroids or biologic therapy could potentially lead to disseminated disease [1]. Chronic eosinophilic airway and lung diseases—such as chronic eosinophilic pneumonia (CEP; eosinophilic pneumonia [EP]), allergic bronchopulmonary aspergillosis (ABPA) or mycosis, eosinophilic bronchitis, and EGPA—demonstrate essential clinical overlap with eosinophilic asthma and CRSwNP. These conditions typically present in adulthood and may occur simultaneously or follow one another in temporal succession [30]. In 2016, Toyoshima et al. proposed the concept of late-onset hypereosinophilic asthma (LHEA) with systemic eosinophilic manifestations (SEMs), as its characteristics differed from CEP, ABPA, and EGPA. LHEA was characterized by the presence of eosinophilic asthma and organ infiltration, without vasculitis, peripheral neuropathy, or cytoplasmic antibodies [31]. These eosinophilic airway/lung diseases respond well to initial treatment with systemic corticosteroids but often recur when corticosteroids are tapered [30].

Eosinophils perform a wide range of functions, from immune regulation and anti-inflammatory activities to the maintenance of tissue homeostasis [22]. Their association with cancer pathogenesis was also reported [32,33,34]. Studies highlight eosinophils’ anti- and pro-tumorigenic roles in tumor microenvironment dynamics [35], suggesting that an anti-tumorigenic role predominates [34]. A Th1 or balanced Th1/Th2 immune response appears to suppress tumors through eosinophils, while an overactive T2 response, which inhibits T1 immunity, promotes tumor progression [34]. The increase in absolute EOS count analyzed by Wang et al. was associated with a lower overall cancer risk, supporting its anti-tumorigenic role, with the exception of intrahepatic bile duct cancer, Hodgkin lymphoma, diffuse large B cell lymphoma, and chronic myeloid leukemia, where an opposite association was observed [32].

### 2.2. Other T2 Conditions

Type 2 mediators, cytokines and immune cells participating in the T2 inflammation response, have been recognized as key contributors to the development of the cluster of diseases established as Type 2-related conditions, which, aside from T2-high asthma, include the following: CRSwNP, prurigo nodularis (PN), AD, and some cases of FA and EoE [1,11,12].

CRSwNP typically presents with nasal obstruction, anosmia or hyposmia, nasal discharge, and sleep disturbances [6]. IL-4, -5, and -13, along with IgE and eosinophils, contribute to maintaining inflammation and facilitating nasal polyp formation. Moreover, endoscopy usually reveals dense, eosinophil-rich mucin with a sticky, “gum-like” consistency with a histopathological image of eosinophil clusters and Charcot–Leyden crystals, serving as key diagnostic markers [11,12]. It is suggested that polypectomy might reduce eosinophilic infiltration into the sinus mucosa by decreasing the endothelial L-selectin ligand levels. In the case report by Gupta et al., a 32-year-old female SA patient underwent nasal endoscopy with polypectomy, which decreased the level of blood EOSs, resolving her HE. The authors emphasize that the procedure alone, without MAbs, does not ensure prevention of recurrence; the patient received benralizumab [36]. Moreover, Tsurumaki et al. have reported the effectiveness of benralizumab in a case of SA with CRSwNP and HE [37]. Cystatin SN, encoded by the CST1 gene, is identified as a key prognostic and predictive marker for CRSwNP, increasing eosinophil activation and IL-5 infiltration [12].

AD affects up to 20% of the pediatric population and up to 5% of adults [6]. It is characterized by heterogeneous itch, skin dryness, and typical lesions such as lichenification. The complex pathophysiology involves skin-barrier dysfunction, microbiome alterations, and immune dysregulation [6,12]. Allergen-stimulated basophils produce leukotriene C_4_, which activates sensory nerves via the cysteinyl leukotriene receptor (CysLTR)2 signaling axis, contributing to acute itch flares. Additionally, deletion of inhibitory kappa β kinase increases C-C motif chemokine ligand (CCL)11 expression by fibroblasts, promoting eosinophilia and shifting the inflammatory response toward a T2 profile in AD [12]. The JAK–signal transducer and activator of transcription (JAK–STAT) signaling pathways mediate the downstream effects of key cytokines implicated in the pathogenesis of AD, including IL-4, -5, -13, -22, -31, and TSLP [12]. Moreover, the JAK2-STAT pathway mediates anti-apoptotic signals in eosinophils in response to GM-CSF and IL-5 [24]. Consequently, oral Janus kinase inhibitors (JAKi’s) have been integrated into therapeutic strategies for this condition [12].

Chronic prurigo nodularis (CNPG) is another skin disorder characterized by persistent pruritus (lasting ≥ 6 weeks) and multiple papules or nodules [38]. CNPG-specific fibroblasts, distinct from those in AD, exhibit high IL-24 and low C-X-C motif ligand (CXCL)14 expression. IL-13 induces IL-24, which downregulates filaggrin via STAT3, impairing the skin barrier. Both CNPG and AD show elevated IL-31 and oncostatin M (OSM), with CNPG also displaying increased neuromedin B [12]. As part of the inflammatory axis, OSM has been linked to asthma severity and reduced lung function. It is a cytokine from the gp130 ligand family, produced by immune cells. In severe asthma, the expression of OSM in isolated blood eosinophils was significantly higher than in controls and patients with CRSwNP, with OSM levels correlating with markers of asthma control loss, including asthma control questionnaire (ACQ) scores and exacerbation frequency [39]. T2 cytokines contribute to itch in AD [12].

FA encompasses a spectrum of immunological mechanisms, from IgE-mediated to non-IgE-mediated [40]. Symptoms range from mild oral tingling to severe, systemic reactions, requiring strict allergen avoidance and, in some cases, the carrying of adrenaline autoinjectors [6]. IgE-mediated food allergies are a growing global concern, particularly in children. Confirmed prevalence of self- or parent-reported FA ranges between 7.6% in children and 10.8% in adults, although the prevalence of clinically proven FA was much lower in studies [6,41]. EoE frequently coexists with IgE-mediated FA, with increasing prevalence in pediatric populations [41], and affects FA patients significantly more often than the general population [40]. Characterized by mucosal barrier dysfunction and progressive tissue remodeling, EoE manifests with gastrointestinal symptoms that vary with age [6]. The success of food elimination diets suggests EoE as a food allergen-driven disease [40].

### 2.3. EGPA and Asthma

EGPA typically progresses through three clinical phases: adult-onset asthma with CRSwNP, peripheral eosinophilia with systemic involvement, and necrotizing vasculitis affecting multiple organs [42]. Eosinophilic asthma is observed in 95–100% of cases and usually precedes vasculitic manifestations by 3 to 11 years. Asthma in EGPA often requires OCSs for symptom control and tends to worsen several months before the onset of systemic disease [43]. Although asthma is a defining feature of EGPA, the relationship between its course and systemic manifestations remains incompletely understood [44]. EGPA should always be considered in patients with blood EOS counts > 1000–1500 cells/µL, eosinophilic asthma, and nasal polyposis with migratory pulmonary infiltrates [45].

Induction of remission in newly diagnosed or relapsing EGPA varies depending on severity and comorbidities. A non-severe case could be approached with a combination of OCSs and mepolizumab, whereas treatments options for severe cases, with organ- or life-threatening involvement, include high-dose OCSs combined with cyclophosphamide (CTX) or rituximab (RTX) monotherapy [10,43,46].

Several case reports have highlighted the potential for EGPA to emerge during or after biologic SA therapy—for instance, during benralizumab treatment, possibly following tapering of systemic corticosteroids that had previously suppressed vasculitis symptoms [37], or shortly after dupilumab cessation [47]. Similarly, Poisson et al. presented a case series of a group of patients suffering from SA or nasal polyps, who switched from mepolizumab/benralizumab medication to dupilumab due to insufficient symptom control. Within six months of the switch, the majority experienced eosinophilia or EGPA relapse [48].

### 2.4. HES and Asthma

The clinical presentation of HES reflects systemic eosinophil-mediated damage and may involve the skin, lungs, gastrointestinal tract, cardiovascular system, and central nervous system, potentially leading to serious complications [9].

Depending on the cause, eosinophilia may be classified as primary/intrinsic (hematological, where eosinophils form part of a neoplastic clone), secondary/reactive (drug- or disease-induced), or idiopathic, which is diagnosed by the exclusion of the former two categories. Each case of HE associated with organ damage may be referred to as HES. Further classification includes specific variants designated by subscripts: HESN (neoplasia), HESR (reactive), HESI (idiopathic), HESFA (familial), and organ-restricted HES [9,24]. In contrast to secondary eosinophilia, primary eosinophilia is associated with myeloid or lymphoid neoplasms associated with tyrosine kinase gene fusions. Such neoplasms involve platelet-derived growth factor receptors (PDGFR)A and PDGFRB, fibroblast growth factor receptor (FGFR)1, pericentriolar material 1 (PCM1)-JAK2, FMS-like tyrosine kinase 3 (FLT3), and tyrosine protein kinase v-abl Abelson murine leukemia viral oncogene homolog 1 (ABL1). Reactive eosinophilia is most commonly related to infections, drug reactions, and collagen vascular diseases [24].

Secondary non-pharmaceutical causes of eosinophilia, not associated with allergies, atopy, or T2 inflammation include the following: Wells syndrome, parasitic and fungal infections, gastrointestinal and rheumatological disorders, vasculitides (e.g., EGPA), EPs, atheroembolic disease, chronic graft-versus-host disease, Gleich syndrome, immune dysregulations, and other non-hematological neoplasms (e.g., solid tumors, lymphomas, leukemias, and systemic mastocytosis) [24].

In a manner akin to the previously described eosinophilia, HES has a predilection for cutaneous manifestations, observed in over 50% of patients, typically presenting as pruritic erythematous macules, papules, plaques, wheals, or nodules [9]. Urticaria and angioedema are characteristic of certain subtypes [9]. Pulmonary involvement occurs in 40–60% of cases and includes symptoms such as cough, wheezing, sputum production, and dyspnea, which may closely resemble bronchial asthma [49]. Such overlap can lead to misdiagnosis, particularly when wheezing or cough are dominant features, especially in children among whom these symptoms are common [50].

In patients with HES, asthma is one of the most frequent accompanying comorbidities, with a prevalence estimated at approximately 45% in Europe [51]. Recognizing HES as a potential differential diagnosis in patients with a clinical presentation of SA with HE, alongside normal spirometry—especially in treatment-refractory cases—is crucial, as early identification may lead to better clinical outcomes [4,50,52]. In a 2021 study by Wei et al., 36 patients with idiopathic and parasite-induced HES, who initially presented asthma-like symptoms, were retrospectively analyzed. OCSs were effective in both groups, while the parasite group required combined deworming medication. Treatment duration was significantly longer for the idiopathic HES group [53]. Cardiovascular complications include heart failure, endocarditis, restrictive cardiomyopathy, arrhythmias, and thromboembolic events due to endomyocardial fibrosis or intracardiac thrombi [9,54]. Riego et al. reported cases of HES onset presenting with asthma-like symptoms, where elevated troponins and eosinophils suggested eosinophilic inflammation of the heart during the course of HES [52]. Gastrointestinal manifestations include dysphagia, weight loss, abdominal pain, nausea, vomiting, and diarrhea. These may result in eosinophilic colitis, enteritis, gastritis, cholangitis, or chronic active hepatitis [9]. Neurological complications range from polyneuropathy and thromboembolic stroke to encephalopathy, with symptoms such as memory loss, dementia, ataxia, seizures, pyramidal signs, and paresthesia reported in cases with central nervous system involvement [55].

The primary goal of HES therapy is to reduce eosinophil counts and prevent organ damage. Treatment options include OCSs, cytotoxic agents, tyrosine kinase inhibitors (TKIs), MAbs, and chemotherapy. OCSs are the first-line therapy (for lymphocyte-variant HE and HES), with prednisone recommended at 1 mg/kg/day, gradually reduced if symptoms improve and eosinophil counts fall below 1500 cells/μL [24,53]. Identifying PDGFRA or PDGFRB rearrangements is critical, as imatinib effectively treats these conditions, whereas pemigatinib is approved for refractory FGFR1-rearranged neoplasms [24].

## 3. Treatment of Hypereosinophilic Asthma

When an underlying cause is not readily identifiable, the management of eosinophilia with pulmonary involvement primarily relies on eosinophil-targeted therapies [24]. However, when eosinophilia is suspected to result from atopic or non-atopic asthma, current World Health Organization (WHO) guidelines emphasize the importance of treating the underlying cause itself [24].

Administration of OCSs and MAbs targeting interleukin IL-5/5R, such as mepolizumab, benralizumab, and reslizumab, is considered in eosinophil-related conditions and for improving asthma symptom control. However, the treatment for all these conditions varies, and its effectiveness strongly depends on a proper identification of the main cause of eosinophilia [1,10,24]. Resolving HE with standard asthma medication is rare; however, such a case was reported by Finley et al. and proves that verifying patients’ adherence and former medication prior to advancing treatment options could be helpful in general management [56]. The escalation of ICS–LABA therapy, phenotype-directed add-ons, or low-dose OCSs should precede the initiation of biologics due to their high cost and limited availability [1,24]. MAbs targeting IL-5 or its receptor have demonstrated efficacy in reducing both blood EOS levels and the frequency of exacerbations [57], while dupilumab was associated with inducing HE rather than treating it [58]. It is not recommended to use dupilumab for patients with current or past blood EOS counts > 1500 cells/μL [4].

The incidence of eosinophilia during dupilumab treatment has been reported to range from 4.1% to 14% [47], with HE affecting approximately one in twenty patients receiving dupilumab for SA in clinical practice [58]. A transient rise in peripheral blood EOS counts typically occurs within the first few weeks of therapy and usually returns to baseline or below by the end of the treatment period. However, in some cases, this elevation may lead to eosinophilic complications. For instance, Gawlewicz-Mroczka et al. reported a case of erythema nodosum (EN) and EP as adverse effects of dupilumab treatment [59]. The proposed mechanism involved impaired eosinophil tissue migration due to IL-4/IL-13 blockade, which affects vascular cell adhesion molecule 1 (VCAM-1) expression on endothelial cells and could contribute to elevated circulating eosinophil levels [59]. Masumoto et al. described dupilumab treatment for SA with mucus plugs that initially showed clinical improvement and normalization of EOS count, but in four months, despite symptom stability, the treatment was followed by rebound eosinophilia [60].

Mepolizumab, the first approved agent in this class, has a broad range of indications and a favorable safety profile [61,62]. The REAL-world effectiveness of mepolizumab In paTIent care—Asthma (REALITI-A) study has presented its initial observations, suggesting that during the first 12 months of treatment for severe eosinophilic asthma, mepolizumab can reduce blood eosinophil counts by 83% [20]. In a phase 3 randomized clinical trial, patients with HES mepolizumab had a significantly reduced blood EOS count by week 2. It was sustained until week 32, with a reduction of 92% from baseline count [63]. The Nucala Effectiveness Study (NEST) showed that after two years of treatment with mepolizumab for SA, the proportion of patients with no clinically significant exacerbations increased from 37.7% to 72%, and the percentage of patients requiring administration of maintenance OCSs for ≥26 weeks decreased from 52.8% to 16.6%, with 82.5% of patients achieving at least a 50% reduction in their OCS dose [64]. Munari et al. have presented mepolizumab as a good alternative for patients with hypereosinophilic SA and CRSwNP previously treated with dupilumab [65]; however, these data do not support verified formal guideline recommendations. In contrast, another SA patient, a 97-year-old woman with peripheral eosinophilia and mucus plugs, failed to respond to mepolizumab but improved significantly upon switching to dupilumab [66].

From early in vitro experiments to phase 1–3 clinical trials, benralizumab has consistently demonstrated efficacy in reducing peripheral blood eosinophil counts by approximately 40% to 61% relative to baseline [67]. The phase 3 randomized controlled Multicenter InSync Randomized Clinical Evaluation (MIRACLE) trial, which focused on Asian patients with eosinophilic SA, along with a retrospective observational study by Pini et al., confirmed both the safety and long-term efficacy of benralizumab. These studies reported improvements across multiple asthma-related clinical outcomes and also highlighted the beneficial effects on symptoms of CRSwNP [68,69]. Matsuno et al. reported effective suppression of blood and ethmoid sinus tissue eosinophils in a patient with hypereosinophilia manifesting after reduction of the OCS dose [70]. Similarly, Just et al. described six pediatric patients with refractory hypereosinophilic asthma, and possibly HES, unresponsive to OCSs, omalizumab, and mepolizumab, in whom benralizumab achieved asthma control and steroid discontinuation [71].

Reslizumab, administered intravenously, also significantly reduced exacerbation rates and OCS use in patients with eosinophilic SA, particularly in patients with high baseline EOS counts (≥400 cells/μL), according to real-world study reports by de Llano et al. and Hashimoto et al. [72,73]. Park et al. described a case of rapid normalization of eosinophilia and improvement of clinical symptoms in imatinib-induced DRESS [74]. Moreover, Chu et al. have highlighted it as a treatment option for non-episodic angioedema with eosinophilia [75].

Together, these biologics offer effective targeted therapy options for patients with eosinophilic SA, particularly those with persistent eosinophilia despite standard treatment.

## 4. Discussion

This review was conducted with the use of academic databases accessible through digital search engines, including PubMed, the Cochrane Library, Google Scholar, ScienceDirect, SpringerLink, and the Wiley Online Library. Search results with key terms such as ‘*severe asthma*’, ‘*hypereosinophilia*’, ‘*hypereosinophilic asthma*’, and ‘*T2-high asthma*’ were thoroughly analyzed and reviewed. The main inclusion criterion was severe asthma accompanied by hypereosinophilia. Works pertaining to the course of SA with diagnosed EGPA or HES according to the recent guidelines were excluded. One case report, Just et al., was included, although diagnosis of HES could not be excluded due to the absence of the updated time-based diagnostic criterion (at least 4 weeks) between two instances of documented marked HE [71]. The standard treatment recommendations, as well as definitions, for asthma and HE and HES were based on GINA reports—for asthma—and the “*World Health Organization and Internal Consensus Classification of eosinophilic disorders: 2024 update on diagnosis, risk, stratification and management*” [1,24,76]. This review includes the most recent data published in the field, with sources no older than six years; however, this exclusion criterion was not applied to studies involving SA with HE, as it is very rare. The most recent report was published in 2024 [48], and the oldest in 2016 [31]. The majority of the publications are in English, with a few in Polish and one in Korean, for which an English abstract was available.

The main limitation of this review is the scarcity and heterogeneity of data on hypereosinophilic asthma, which still remains an extremely rare clinical entity in the literature. This may be partly attributable to the evolving diagnostic criteria for HES, which have been refined to include such criteria as time in between the measurements and specific types of tissue damage resulting from eosinophilic infiltration [24,76]. The exclusion of EGPA in cases of SA with HE appears increasingly feasible based on the American College of Rheumatology and European League Against Rheumatism (ACR/EULAR) classification criteria; however, it is important to note that these criteria are intended for classification rather than for diagnostic purposes and do not constitute formal diagnostic standards for EGPA [10,45]. An additional diagnostic challenge lies in the narrow boundaries between these disease entities and other eosinophilic inflammatory conditions of the respiratory tract. Hypereosinophilic asthma may evolve into HES or, during the course of treatment, reveal EGPA. Differentiating between HES and EGPA can be particularly difficult in patients presenting predominantly with respiratory symptoms [77]. Asthma-like symptoms are frequently observed in diseases such as EGPA and HES; therefore, rigorous differential diagnosis and the application of appropriate exclusion criteria are critical in proper diagnosis.

No cases of comorbidity-free asthma have been identified. Hypereosinophilic asthma has been described in seven publications (with one exception suggesting a possible HES diagnosis), which are summarized and presented in Table 1, ‘*Cases of severe hypereosinophilic asthma*’.

Abbreviations which were not introduced in the text but are present in the Figures and Tables are as follows: angiotensin-converting-enzyme inhibitor (ACEI), antiretroviral drug (ARV), anticonvulsant drug (AED), artery thrombosis (Ath), bronchothermoplasty (BTP), chronic urticaria (CU), chronic spontaneous urticaria (CSU), deep vein thrombosis (DVT), eosinophilic gastroenteritis (GE), eosinophilic gastrointestinal disease (EGID), eosinophilic sinusitis (ES), erythropoietin (EPO), gastroesophageal reflux disease (GERD), hydroxyurea (HU), immune checkpoint inhibitor (ICI), immunomodulator (IM), kinase inhibitor (KI), leukotriene receptor antagonist (LTRA), nasal polyposis (NP), otitis media (OM), phosphodiesterase 5 (PDE5) inhibitor, proton-pump inhibitor (PPI), pulmonary embolism (PE), vernal keratoconjunctivitis (VKC), warfarin (WARF), and xanthine oxidase inhibitor (XOI).

**Figure 1:**
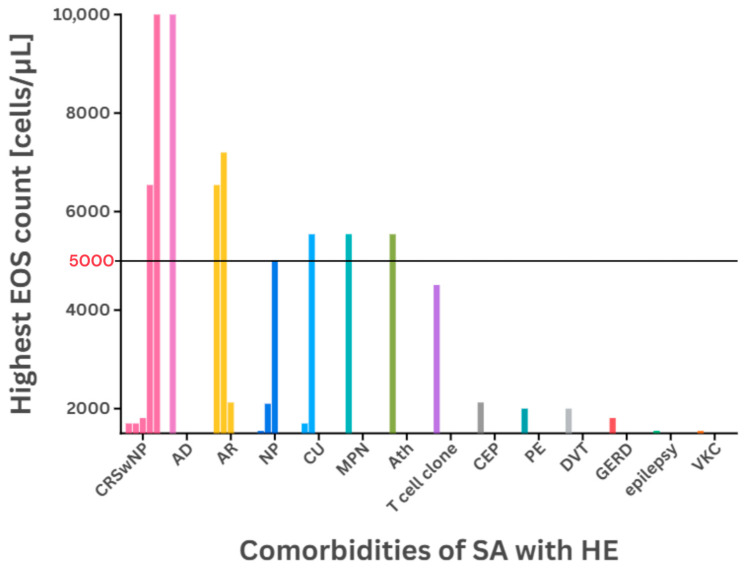
Comorbidities of SA with HE and diseases associated with hypereosinophilia. The grouped column chart represents the highest EOS counts reported in the literature for each coexisting condition. The number of columns reflects the frequency of occurrence (e.g., 5 CRSwNP, 1 AD, etc.). The chart was divided by a horizontal line into two sections: moderate (≤5000) and severe (>5000) eosinophilia. Two records (CRSwNP and AD) exceeded the scale with the highest EOS count 40,400 cells/µL in one pediatric patient [71].

Each case involved at least one comorbidity, most frequently associated with other eosinophilic disorders with T2 inflammation, possibly contributing to asthma-induced eosinophilia. The prevalence of comorbidities reported in the reviewed cases is illustrated in Figure 1, ‘*Comorbidities of SA with HE and diseases associated with hypereosinophilia*’.

Severe eosinophilia (>5000 cells/µL) was observed in patients with Ath, CU, CRSwNP, AD, AR, and MPN. On a global scale, approximately 26.70% of asthma patients experience SA, 30.99% have eosinophilic asthma, 48.95% have concurrent AR, and 7.0–25.40% suffer from NP [78]. The overall view on the prevalence of diseases associated with eosinophilia reported by the retrospective study of Yu et al. confirms the rarity of the reviewed cases and their comorbidities. Allergies were found to cause mild eosinophilia, and only about 10.8% of cases resulted in HE [79]. CRSwNP and AR were the most common conditions observed. CEP was found to be able to cause severe or moderate HE, with or without coexistence of SA. Similarly, EGID was reported to cause severe eosinophilia. These data may be useful for defining priorities in subsequent studies. There is a need for further research to more closely investigate the correlation between asthma comorbidities and the manifestation of hypereosinophilia or HESR, secondary to asthma and T2-related conditions. The prevalence of non-asthma diseases associated with hypereosinophilia is presented in Table 2, ‘*Prevalence of conditions with predominant eosinophilic involvement*’.

The resolution of severe HE with standard asthma management is marginally rare and only one case so far was presented by Finley et al., where a patient’s EOS count reduced from 7200 cells/µL to 1200 cells/µL within two weeks of budesonide/formoterol treatment, and then down to 400 cells/µL 6 weeks after [56]. Apart from patients’ adherence, it is also important to verify their past and current medications and diagnoses.

The lack of specific guidance on the management of SA with HE in international asthma guidelines is considered another limitation. Published data lack consensus and concordance on diagnosis, pharmacological treatment, and overall management of this condition. In most cases, therapeutic decisions were based on individual clinical assessments, due to insufficiently specific recommendations that fail to account for associated comorbidities, current EOS counts, the presence of complications, and preventive strategies. The available evidence derives primarily from case reports and retrospective observational studies. There is a need for further research in this area to better understand the nature of the condition and to enable the implementation of appropriate strategies that would facilitate accurate diagnosis and effective treatment. Potential asthma medications relevant to the reviewed cases are listed in Table 3, ‘*Asthma medications associated with HE*’. The table includes the reported impact of dupilumab on the development of HE.

**Figure 2:**
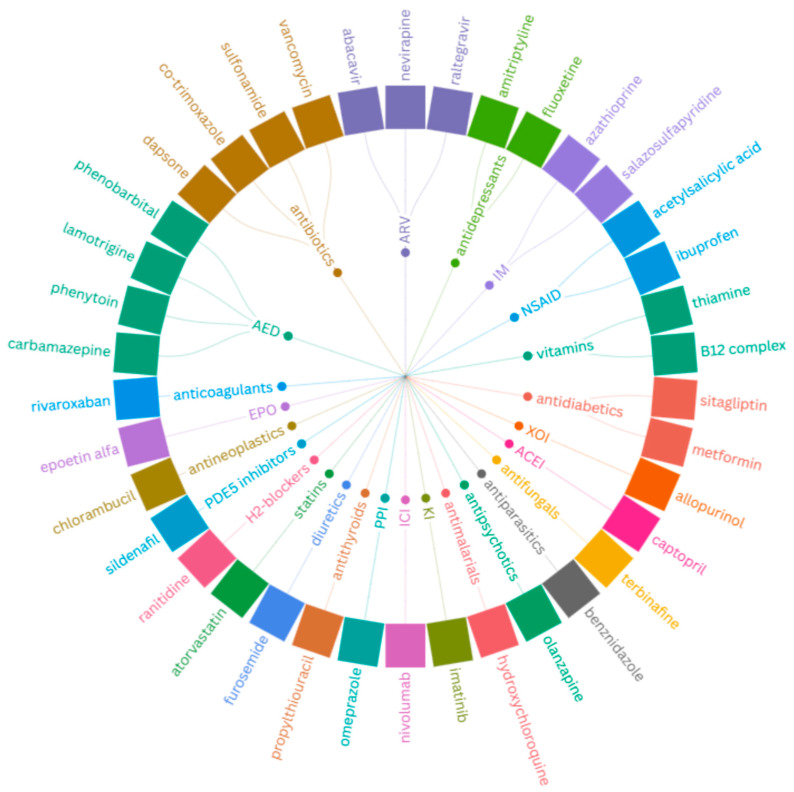
Drugs inducing eosinophilia. The radial chart illustrates pharmacological classes (inner ring) and their representatives (outer ring) that have been associated with eosinophilia. Several of these medications are commonly used to treat comorbid conditions in patients with SA. Verifying current medications and patient adherence are essential in the diagnostic and differential process of SA with HE.

In cases where medication does not bring satisfactory results, reevaluation might be necessary. The Re-Qualification of the asthma patient on Biologic therapy (ReQualBi) study has highlighted the importance of verifying prior diagnoses in patients with SA in whom HE persists despite therapy with anti-IL-5/5R MAbs, or in whom continued eosinophilia persists despite daily OCSs. These patients require rediagnosis and confirmation of SA without another underlying cause of HE (including drugs) before being requalified for a switch in biologic therapy [84]. Drugs which were reported to induce eosinophilia were collected based on Criado et. al. and Chen et al. publications and are shown in Figure 2, ‘*Drugs inducing eosinophilia*’ [85,86].

Non-asthma-related conditions potentially influencing asthma management and medication range from benign to life-threatening and must not be overlooked. Although broadly applicable medications with demonstrated efficacy in several related conditions—such as mepolizumab—are available, the cornerstone of managing persistent HE remains a thorough reassessment of the diagnosis and previously applied treatments.

## 5. Conclusions

Hypereosinophilia occurring in the context of severe asthma is an exceptionally rare condition that requires careful differentiation from other eosinophilic disorders such as HES and EGPA, as these entities differ significantly in terms of pathogenesis, prognosis, and management strategies. While respiratory symptoms are rarely the sole manifestation of hematologic diseases, asthma may present as part of their clinical spectrum and should be considered in the differential diagnosis. A diagnosis of hypereosinophilic asthma may be established by exclusion, following the confirmation of persistent eosinophilia (lasting ≥ 4 weeks), evidence of organ damage, and features of vasculitis, with special attention paid to the systematic reassessment of patients with ongoing hypereosinophilia despite having received anti-IL5/5R biologic therapy, and in patients with persistent eosinophilia while remaining on OCSs. These patients should undergo rediagnosis of severe asthma before being reconsidered for further SA therapy. SA with HE has been observed in patients with comorbidities, where overlapping mechanisms contribute to elevated eosinophil counts. The highest EOS counts were observed most frequently in cases with AR, CRSwNP, and NP comorbidities. Current treatment options are primarily limited to systemic corticosteroids and biologic agents targeting IL-5/5R. The use of dupilumab (anti-IL-4Rα) in patients with marked peripheral HE remains controversial and is not recommended. Further research is warranted to define diagnostic criteria and optimize therapeutic approaches for patients with hypereosinophilic asthma.

## 6. Summary

Asthma is a heterogeneous condition typically associated with airway hyperresponsiveness and chronic inflammation. Approximately 3–10% of patients have severe asthma, 60% of whom exhibit an allergic or non-allergic, eosinophilic endotype characterized by T2 inflammation, also known as Th2-high asthma. SA accompanied by HE remains a rare clinical entity, identified in approximately 0.3% of cases, and poses diagnostic and therapeutic challenges due to the lack of established treatment guidelines and limited representation in clinical trials. This review addresses such presentations in patients who do not fulfill the WHO diagnostic criteria for HES or EGPA.

T2-high asthma involves both adaptive (allergen-driven) and innate (e.g., virus-induced) immune pathways, leading to the activation of eosinophils, mast cells, Th2 cells, and ILC2s. The allergic (early-onset) phenotype is linked to atopy and elevated IgE, while the eosinophilic (late-onset) variant is often resistant to ICSs and associated with conditions like CRSwNP or EGPA. Recognition of T2-high asthma relies on biomarkers such as blood and sputum EOS counts, FeNO, and often IgE overproduction (in atopic endotype), while treatment focuses on ICS-LABA MART regimens and specific add-ons depending on clinical presentation, such as maintenance OCSs or MAbs targeting key cytokines (e.g., IL-4, IL-5/5R, and TSLP).

T2 inflammation is mediated by either the adaptive or innate immune system. It underlies a spectrum of diseases apart from asthma, including CRSwNP, AD, PN, FA, and EoE—some of which are part of the atopic march and typically first manifest during infancy. These conditions share overlapping mechanisms, such as epithelial barrier dysfunction, chronic inflammation, and EOS infiltration, leading to diverse clinical manifestations ranging from respiratory symptoms to skin lesions and gastrointestinal involvement. They can coexist with asthma and contribute to eosinophilia. Therapeutic strategies for non-asthma T2 conditions may target shared molecular pathways, including JAK–STAT signaling and IL-5-mediated eosinophil activation, to improve disease control and reduce recurrence.

Hypereosinophilia (blood EOS count > 1500 cells/µL) may be symptomatic or asymptomatic and can result either from primary/clonal disorders, such as MPNs, or secondary inflammatory causes (e.g., parasitic infections, allergic diseases, EGPA, drugs, and cancer). If none of the previous causes is identified, idiopathic HES may be diagnosed by exclusion. In cases of hypereosinophilic asthma, differential diagnosis from other eosinophilic lung diseases (e.g., EP and ABPA) is essential, as inappropriate treatment may be ineffective and worsen the patient’s condition.

EGPA is a complex disease typically evolving from adult-onset asthma and nasal polyposis to systemic eosinophilia and necrotizing vasculitis. Treatment requires individualized approaches, with caution in differential diagnosis, as its clinical presentation may mimic other conditions such as severe eosinophilic asthma or HES. HES represents a heterogeneous group of disorders characterized by persistent eosinophilia and organ damage, most commonly affecting the skin, lungs, heart, and nervous system. It is classified as primary (clonal), secondary (reactive), or idiopathic. Treatment is tailored to the underlying cause, with the primary goal of reducing eosinophil counts to prevent complications. OCSs are the first-line therapy, while TKIs such as imatinib or pemigatinib are used in genetically defined subtypes with PDGFRA, PDGFRB, or FGFR1 rearrangements.

In the treatment of eosinophilia with pulmonary involvement, identifying the underlying cause is crucial. In particular, patients with SA who continue to present with persistent hypereosinophilia after receiving anti IL-5/5R-targeted biologic treatment or eosinophilia despite maintenance OCSs, should undergo a re-evaluation of the SA diagnosis before being requalified for further SA asthma treatment. Dupilumab has demonstrated efficacy in treating T2-high allergic asthma but carries a risk of transient or complicated eosinophilia and is not recommended in patients with current or prior hypereosinophilia >1500/μL. IL-5/5R targeting MAbs, such as mepolizumab and benralizumab, which have shown favorable safety profiles and effectiveness in both clinical trials and real-world settings, including patients with hypereosinophilic syndrome.

## Figures and Tables

**Table 1:** Cases of severe hypereosinophilic asthma. Summarized information from case reports and studies reporting cases of SA with HE, which were not diagnosed as HES or EGPA (with one possible exception ^4^). Presented cases are rare and have no standardized treatment. Among medications administered with success are OCSs (e.g., prednisone), MAbs (omalizumab, benralizumab, and mepolizumab), IMs (e.g., cyclosporine), and standardized asthma and NP treatment.

Patient Details [(M) Male/(F) Female (Age)]	Highest EOS Count [cells/µL]	Comorbidities	Previous Treatment	Administered Medication	[Source]
M (51)	>1700	CRSwNP	OCS, omalizumab, BTP	benralizumab	[37]
M (55)	7200	AR	SABA, OCS	ICS/LABA, LTRA, SABA	[56]
F (36)	5540	CU, MPN	HU, WARF, ICS	OCS	[7]
F (54)	4510	T cell clone	ICS	OCS	
M (62)	2000	PE, DVT	ICS, WARF	OCS	
4 M 11 F (mean 43.7)	- ^1,2^	EP, ES, GE, OM	- ^1^	OCS	[31]
F (32)	6540	CRSwNP, AR	- ^1^	polypectomy	[36]
F (22)	2125	CEP, AR	ICS/LABA, OCS	OCS, dupilumab	[60]
F	1810	CRSwNP, GERD	OCS, omalizumab, mepolizumab	OCS, omalizumab, benralizumab	[71] ^3,4^
M	1550	NP, VKC, epilepsy	OCS, omalizumab, mepolizumab	OCS, omalizumab, mepolizumab, benralizumab	
M	5000	NP	OCS, omalizumab, mepolizumab	OCS, omalizumab, mepolizumab, benralizumab	
M	1700	CRSwNP, CU	OCS, omalizumab, mepolizumab	OCS, omalizumab, mepolizumab, benralizumab	
M	40,400	CRSwNP, AD	OCS, omalizumab, mepolizumab	OCS, IM, omalizumab, mepolizumab, benralizumab	
F	2100	NP	OCS, omalizumab, mepolizumab	OCS, omalizumab, mepolizumab, benralizumab	

^1^ No data, ^2^ criteria of HE met, ^3^ mean age: 5.5 y.o., ^4^ HES could not be excluded.

**Table 2:** Prevalence of conditions with predominant eosinophilic involvement. This table presents non-asthma conditions associated with marked EOS counts in peripheral blood. The column labels “Mild”, “Moderate”, and “Severe” refer to severity of eosinophilia. The prevalence data are based on a retrospective study by Yu et al. [79]. The symbol “+” indicates the number of reviewed cases in which the asthma comorbidity matched the condition from the observational study. Conditions not identified as comorbidities were marked with “-“, including EGPA and HES.

Condition	Mild (%)	Moderate (%)	Severe (%)	Asthma Cases with HE
**Allergic diseases**	59.8	10.8	- ^1^	+++
**Parasitic infestation**	22.7	29.7	14.3	-
**Drug allergy**	13.4	37.8	14.3	-
**HES**	1.0	8.1	21.4	-
**EGPA**	1.0	2.7	28.6	-
**ABPA**	1.0	2.7	7.1	-
**CEP**	- ^1^	8.1	7.1	+
**EGID**	1.0	- ^1^	7.1	+

^1^ No data.

**Table 3:** Asthma medications associated with HE. Among the most frequently recommended drugs in conditions associated with HE are MAbs targeting IL-5/5R and OCSs. Mepolizumab is currently recommended for the widest range of indications. Benralizumab is efficient in reducing EOS count to 0 cells/μL. The use of dupilumab is not recommended for cases with HE, carrying risk of transient or complicated eosinophilia.

	Drug	Used in	Efficacy		[Source]
**medication resolving HE**	mepolizumab	SA, CRSwNP, EGPA, HES	SA	83% reduction at 12 months ^1^	[63]
			HES	92% reduction at 32 weeks	
	benralizumab	SA, HES, EoE, CRSwNP ^3^	HES	down to 0 cells/μL by week 4 ^2^	[25]
			612.78 cells reduction at 12 months		[80]
	reslizumab	HES ^4^	5438 to <500 cells/μL at 21 months		[81]
	omalizumab	HESI	4310 to 1000 cells/μL at 17 months		[82]
			7010 to 2230 cells/μL at 3 months		
	OCS	HESI	2100 to <500 cells/μL at 6 months ^5^		[83]
**asthma medication resulting in HE**	dupilumab	SA, CSU	280 to 310 cells/μL in 4 weeks ^6^		[47]
			400 to 4900 cells/μL in 4 weeks		[58]
			500 to 2000 cells/μL in 16 weeks		
			600 to 9000 cells/μL in 16 weeks		
			800 to 2500 cells/μL in 16 weeks		
			50 to 5000 cells/μL in 16 weeks		
			300 to 2000 cells/μL in 6 months ^6^		
			750 to 4880 cells/μL in >2 months ^7^		[55]

^1^ REALITI-A, ^2^ SIROCCO, ^3^ or without NP, ^4^ lymphocyte variant of HES, ^5^ with heart failure medication, ^6^ developed EGPA, ^7^ developed EN and EP.

## Abbreviations

The following abbreviations are used in this manuscript:

ABL1	v-abl Abelson murine leukemia viral oncogene homolog 1
ABPA	allergic bronchopulmonary aspergillosis
ACEI	Angiotensin-converting-enzyme inhibitor
ACQ	Asthma Control Questionnaire
ACR/EULAR	American College of Rheumatology and European League Against Rheumatism
AED	Anticonvulsant drug
AD	atopic dermatitis
ARV	antiretroviral drug
AR	allergic rhinitis
Ath	artery thrombosis
ATS	American Thoracic Society
BTP	bronchothermoplasty
CEL	chronic eosinophilic leukemia
CCL	C-C motif chemokine ligand
CEP	chronic eosinophilic pneumonia
CNPG	chronic prurigo nodularis
COX-1	cyclooxygenase-1
CRSwNP	chronic rhinosinusitis with nasal polyps
CTX	cyclophosphamide
CU	chronic urticaria
CSU	chronic spontaneous urticaria
CXCL	C-X-C motif ligand
CystLTR	cysteinyl leukotriene receptor
DRESS	drug reaction with eosinophilia and systemic symptoms
DVT	deep vein thrombosis
EGID	eosinophilic gastrointestinal disease
EGPA	eosinophilic granulomatosis with polyangiitis
EN	erythema nodosum
EOS	eosinophil
EoE	eosinophilic esophagitis
EP	eosinophilic pneumonia
EPO	(human) erythropoietin
ERS	European Respiratory Society
ES	eosinophilic sinusitis
ET	essential thrombocytopenia
FA	food allergy
FeNO	fractional exhaled nitric oxide
FGFR	fibroblast growth factor receptor
FLT3	FMS-like tyrosine kinase 3
GE	eosinophilic gastroenteritis
GERD	gastroesophageal reflux disease
GINA	Global Initiative for Asthma
GM-CSF	granulocyte-macrophage colony-stimulating factor
HE	hypereosinophilia
HES	hypereosinophilic syndrome
HESN	HES neoplasia
HESR	HES reactive
HESI	HES idiopathic
HESFA	HES familial
HU	hydroxyurea
ICI	immune checkpoints inhibitor
ICS	inhaled corticosteroid
IL	interleukin
IL-5R	interleukin-5 receptor
ILC	innate lymphoid cell
IM	immunomodulator
JAK	Janus kinase
JAKi	Janus Kinase inhibitor
KI	kinase inhibitor
KIT	KIT proto-oncogene, encoding tyrosine kinase receptor
LABA	long-acting beta^2^-agonist
LHEA	late-onset hypereosinophilic asthma
LTRA	leukotriene receptor agonist
MAb	monoclonal antibody
MART/SMART	maintenance and reliever therapy/single-inhaler MART
MIRACLE trial	Multicenter InSync Randomized Clinical Evaluation trial
MPN	myeloproliferative neoplasm
N-ERD	NSAID-exacerbated respiratory disease
NEST	The Nucala Effectiveness Study
NP	nasal polyposis
NSAID	Non-steroidal anti-inflammatory drug
OCS	oral corticosteroid
OM	otitis media
OSM	oncostatin M
PCM1	pericentriolar material 1
PDE5	phosphodiesterase 5
PDGFR	platelet-derived growth factor receptor
PE	pulmonary embolism
PN	prurigo nodularis
PPI	proton-pump inhibitor
PV	polycythemia vera
REALTI-A	The REAL-world effectiveness of mepolizumab In paTIent care—Asthma
ReQualBi study	Re-Qualification of the asthma patient on Biologic therapy study
RTX	rituximab
SABA	short-acting β2-agonist
SA	severe asthma
SEM	systemic eosinophilic manifestation
STAT	signal transducer and activator of transcription pathway
TKI	tyrosine kinase inhibitor
TSLP	Thymic stromal lymphopoietin
T2	Type 2
VCAM-1	vascular cell adhesion molecule 1
VKC	vernal keratoconjunctivitis
WARF	warfarin
WBC	white blood cell
WHO	World Health Organization
XOI	xanthine oxidase inhibitor
